# A Class Representative Model for Pure Parsimony Haplotyping under Uncertain Data

**DOI:** 10.1371/journal.pone.0017937

**Published:** 2011-03-25

**Authors:** Daniele Catanzaro, Martine Labbé, Luciano Porretta

**Affiliations:** 1 Graphes et Optimisation Mathématique (G.O.M.), Computer Science Department, Université Libre de Bruxelles (U.L.B.), CP 210/01, Brussels, Belgium; 2 Graphes et Optimisation Mathématique (G.O.M.), Computer Science Department, Université Libre de Bruxelles (U.L.B.), CP 210/01, Brussels, Belgium; 3 Graphes et Optimisation Mathématique (G.O.M.), Computer Science Department, Université Libre de Bruxelles (U.L.B.), CP 210/01, Brussels, Belgium; Aarhus University, Denmark

## Abstract

The Pure Parsimony Haplotyping (PPH) problem is a NP-hard combinatorial optimization problem that consists of finding the minimum number of haplotypes necessary to explain a given set of genotypes. PPH has attracted more and more attention in recent years due to its importance in analysis of many fine-scale genetic data. Its application fields range from mapping complex disease genes to inferring population histories, passing through designing drugs, functional genomics and pharmacogenetics. In this article we investigate, for the first time, a recent version of PPH called *the Pure Parsimony Haplotype problem under Uncertain Data* (PPH-UD). This version mainly arises when the input genotypes are not accurate, i.e., when some single nucleotide polymorphisms are missing or affected by errors. We propose an exact approach to solution of PPH-UD based on an extended version of Catanzaro *et al.* [1] class representative model for PPH, currently the state-of-the-art integer programming model for PPH. The model is efficient, accurate, compact, polynomial-sized, easy to implement, solvable with any solver for mixed integer programming, and usable in all those cases for which the parsimony criterion is well suited for haplotype estimation.

## Introduction

The human genome is divided in 23 pairs of chromosomes thereof, one copy is inherited from the father and the other from the mother. When a nucleotide site of a specific chromosome region shows a variability within a population of individuals then it is called *Single Nucleotide Polymorphism* (SNP). Specifically, a site is considered a SNP if for a minority of the population a certain nucleotide is observed (called the least frequent allele) while for the rest of the population a different nucleotide is observed (the most frequent allele) [2]. The least frequent allele, or *mutant type*, is generally encoded as ‘1’, as opposed to the most frequent allele, or *wild type*, generally encoded as ‘0’ [3]. A *haplotype* is a set of alleles, or more formally, a string of length 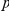 over an alphabet 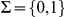 [4]. Haplotypes represent a fundamental source of information for disease association studies. In fact, over 90% of sequence variation among individuals is due to common variant sites, most of which arose from single historical mutation events on the ancestral chromosome [5]. Hence, in a group of people affected by a disease, the SNPs causing or associated with the disease will be enriched in frequency compared with the corresponding frequencies in a group of unaffected individuals. This observation was of considerable assistance, for example, in the identification of the genes responsible for type 1 diabetes [6]–[8], type 2 diabetes [9], [10], Alzheimer's disease [11], deep vein thrombosis [12], inflammatory bowel disease [13]–[15], hypertriglyceridaemia [16], schizophrenia [17], asthma [18], stroke [19], myocardial infarction [20], cystic fibrosis and diastrophic dysplasia [21], [22].

Extracting haplotypes from a population of individuals is not an easy task. In fact, the current molecular sequencing techniques only provide information about the conflation of the paternal and maternal haplotypes of an individual (also called *genotype*) rather than haplotypes themselves [23]. When the family-based genetic information of a population is available, haplotypes can be retrieved experimentally [24]. However, the experimental approach is generally laborious, cost-prohibitive, requires advanced molecular isolation strategies [25], and sometimes not even possible [26]. In absence of a family-based genetic information, a valid alternative to the experimental approach is provided by computational methods which estimate, by means of specific criteria, haplotypes from the set of genotypes extracted from a population of individuals.

A genotype can be formally defined as a string of length 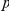 over an alphabet 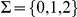, where the symbols ‘0’ or ‘2’ denote homozygous sites (of wild and mutant type, respectively) and the symbol ‘1’ denotes heterozygous sites. As an example, the sequence 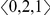 encodes a genotype in which: the first SNP is homozygous of wild type; the second SNP is homozygous of mutant type; and finally the third SNP is heterozygous. A genotype is said to be *degenerate* if it does not contain ‘1’s. A genotype 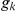 is said to be *resolved* from a pair of haplotypes 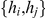, in symbols 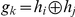, if the 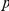-th entry of 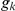, denoted as 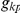, is equal to the sum of the 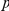-th entries of 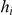 and 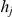, denoted as 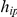 and 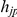, respectively. For example, the genotype 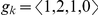 is resolved from 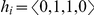 and 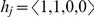. Haplotyping a set of genotypes 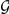 means finding the set of haplotypes resolving 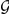.

It is worth noting that, given a genotype and denoted 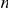 as the number of its heterozygous sites, there exist 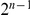 possible haplotypes that may resolve it [26]. As an example, genotype 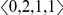 may be resolved from either the pair of haplotypes 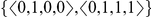 or the pair 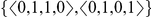. This fact involves as a necessary consequence the use of a criterion to select pairs of haplotypes among plausible alternatives. Gusfield [27] and Wang and Xu [28] observed that the number of distinct haplotypes existing in a large population of individuals is generally much smaller than the overall number of distinct genotypes observed in that population. This insight has suggested that, for low-rate recombination genes at least, the criterion of minimizing the overall number of haplotypes necessary to resolve a set of genotypes may have good chances to recover the biological haplotype set. This criterion, formally introduced by Gusfield [27], is known as *the pure parsimony criterion of haplotype estimation* and was of considerable assistance, for example, in the identification of the genes responsible for psoriasis and severe alopecia areata [2]. Haplotyping a set of genotypes under the parsimony criterion involves solving an optimization problem, called *the Pure Parsimony Haplotyping (PPH) problem*, that can be stated as follows:

**Problem.** *The Pure Parsimony Haplotyping (PPH) problem*

*Given a set* 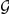 *of* 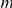 *non-degenerating genotypes, having* 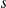 *SNPs each, find the minimum set* 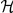 *of haplotypes such that for each genotype* 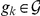 *there exists a pair of haplotypes* 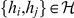 *resolving* 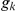 *.*

As an example, an instance of PPH and the corresponding solution is shown in Table 1.

**Table 1:** Graphical representation of an instance of PPH and the corresponding solution.

Instance of PPH				
Genotypes	SNPs			
Genotype 1	2	1	1	2
Genotype 2	1	0	1	1
Genotype 3	1	0	2	2
Genotype 4	2	0	1	1
Genotype 5	2	1	0	1

Solution				
Haplotypes	SNPs			
Haplotype 1	1	0	1	1
Haplotype 2	1	1	0	1
Haplotype 3	0	0	1	1
Haplotype 4	1	0	0	0

PPH is known to be polynomially solvable when each genotype has at most two heterozygous sites [29], and 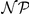-hard when each genotype has at least three heterozygous sites [26].

Recently, Brown and Harrower [30] introduced an interesting version of PPH called *the Pure Parsimony Haplotype problem under Uncertain Data* (PPH-UD). This version mainly arises when the input genotype set 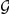 is not accurate, i.e., when some SNPs are missing or affected by errors, a situation that often occurs in practice. In this case, the input of the problem may include also a binary matrix 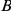, called *the error mask matrix*, whose generic entry 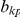 is equal to 1 if the 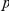-th SNP of genotype 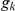 is uncertain (i.e., missing or affected by an error), and 0 otherwise. When a given SNP is uncertain its actual value could significantly deviate from its true value. For example, the true value of a wild type homozygous SNP affected by uncertainty could be homozygous of mutant type or even heterozygous. Similarly, the true value of a heterozygous SNP affected by uncertainty could be homozygous of wild or mutant type. The presence of uncertain data modifies the standard definition of resolution for a genotype. Specifically, Brown and Harrower [30] stated that when uncertainty occurs in the input data a genotype 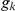 is resolved by a pair of haplotypes 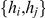 if 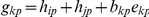, for all SNPs 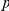, being 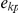 a integer variables assuming values in the set 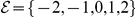. Brown and Harrower [30] described an integer programming model able to tackle instances of PPH affected by uncertain data. Unfortunately, the authors did not offer experimental evidence of the performances of their model due to its unpractical runtimes. In this article we address this critical issue by introducing a possible integer linear programming model to solve exactly instances of PPH-UD. The model is based on an extension of Catanzaro *et al.* [1] Class Representative Model (CRM), currently one of the best integer programming model for PPH described in the literature. The model that we propose is efficient, compact, polynomial-sized, easy to implement, solvable with any solver for mixed integer programming, and usable in all those cases for which the parsimony criterion is well suited for haplotype estimation.

## Methods

As shown in Catanzaro *et al.* [1], any feasible solution of PPH induces a family of subsets of genotype such that: (i) each subset represents one unique haplotype with elements in the subset being genotypes carrying the haplotype, (ii) each genotype belongs to exactly two subsets, and (iii) every pair of subsets intersects in at most one genotype. This principle can be exploited also when dealing with PPH-UD. Specifically, let associate an index to each subset 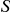 of genotypes induced by a haplotype 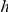. If 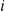 is the smallest index of a genotype belonging to 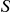, then 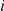 is the index associated to 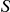 and the subset will be denoted as 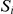. Since each genotype 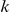 belongs to exactly two subsets (as it must be explained by exactly two haplotypes) it may happen that 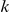 is itself the genotype with smallest index in both subsets. In this case a dummy genotype 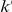 is added, and the subset 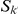 is created. As an example, one can imagine that the haplotype 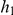 induces the subset 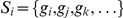, 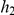 induces the subset 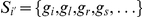, 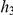 induces the subset 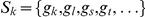, and so on. We remark that the index 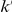 can be considered only if 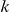 was previously used, i.e., if the subset 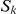 already exists.

Since at most 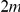 haplotypes are necessary to resolve 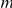 genotypes [26], then the indices 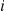 of the subsets 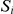 can vary inside the index set 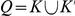, where 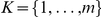 and 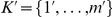. Assume that an order is defined on 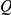 in such a way that 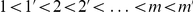. Define 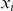, 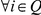, as a decision variable equal to 1 if, in the solution, there exists a haplotype inducing a subset 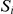 of genotypes whose smallest index genotype is 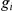, and 0 otherwise. Denote 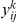, 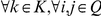, as a decision variable equal to 1 if genotype 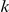 belongs to the subsets 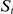 and 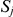, and 0 otherwise. Denote 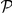 as the set of the input SNPs and 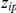, 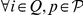, as a decision variable equal to 1 if the haplotype inducing the subset 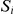 of genotypes has such a value at 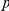-th site, and 0 otherwise. Variables 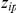 describe explicitly the haplotypes of the solution.

For every non-null entry of the error mask matrix 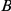 denote 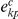 as a decision variable accounting for the difference between the value of 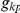 and the true underlying value. Specifically, 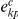 is equal to 1 if the 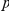-th entry of genotype 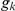 is corrected with a value 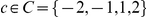, and 0 otherwise. Finally, let 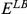 and 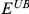 be a lower and an upper bounds on the overall number of errors in 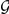. Then, the following model is a valid formulation of PPH-UD:

**Formulation.** *Class Representative Model (CRM) for PPH-UD*

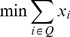
(1.1)

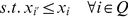
(1.2)

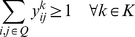
(1.3)

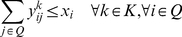
(1.4)

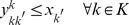
(1.5)

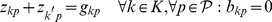
(1.6)

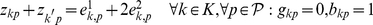
(1.7)

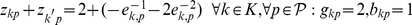
(1.8)

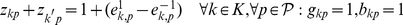
(1.9)

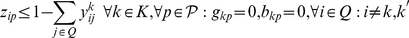
(1.10)

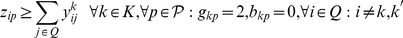
(1.11)

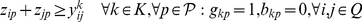
(1.12)

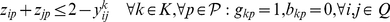
(1.13)

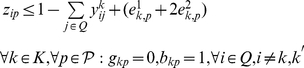
(1.14)

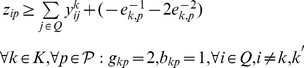
(1.15)

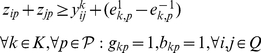
(1.16)

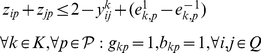
(1.17)

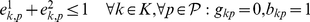
(1.18)

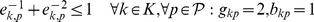
(1.19)

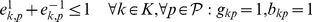
(1.20)

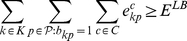
(1.21)

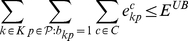
(1.22)

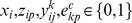
(1.23)The objective function (1.1) represents the number of distinct haplotypes or equivalently the cardinality of 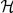. Since the index 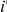 is considered only if 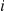 is already used, constraints (1.2) implies that if the haplotype 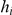 is not used, then 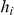 should not be used. Constraints (1.3) impose that each genotype 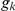 must belong to exactly two subsets 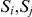, and constraints (1.4) force 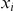 to be 1, i.e., to take haplotype 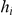 into account, if some genotype 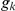 is resolved by 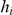. Constraints (1.5) are a consequence of the definition of the dummy genotype 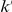. Actually, they constitute a special version of constraints (1.4) when genotype 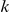 is resolved by haplotype 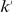. Constraints (1.6) impose the sum operation among haplotypes in absence of uncertainty. Constraints (1.7–1.9) translate the sum operation among haplotypes when uncertainty occurs in the input data. Specifically, constraints (1.7) account for the correction imposed on the 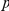-th SNP of haplotypes 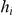 and 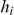 when 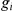 has its 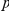-th SNP equal to 0. In this case, two situations may occur at the 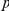-th SNP: either no correction is performed, or a correction is performed by setting to 1 one between 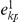 and 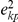. If a correction is performed and 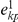 is set to 1 at the 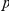-th SNP then one haplotype will be homozygous of wild type and the other of mutant type. On the contrary, if 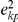 is set to 1 then both haplotypes will be homozygous of mutant type. Constraints (1.8) account for the correction imposed on the 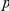-th SNP of haplotypes 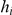 and 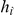 when 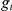 has its 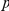-th SNP equal to 2. Similarly to constraints (1.7), also in this case two situations may occur: either no correction is performed, or a correction is performed by setting to 1 one between 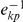 and 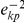. If a correction is performed and 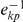 is set to 1 at the 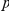-th SNP then one haplotype will homozygous of wild type and the other of mutant type. On the contrary, if 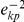 is set to 1 then both haplotypes will be homozygous of wild type. Finally, constraints (1.9) account for the correction imposed on the 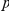-th SNP of haplotypes 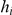 and 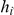 when 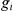 has its 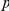-th SNP equal to 1. If a correction is performed and 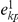 is set to 1 at the 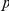-th SNP then both haplotypes will be homozygous of mutant type. On the contrary, if 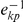 is set to 1 then both haplotypes are homozygous of wild type. Constraints (1.10) establish the relations between variables 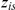 and 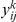 in absence of uncertainty. Specifically, they force the 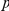-th site of the haplotype 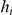 to be equal to 0 when at least one genotype 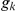, whose 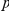-th entry equal to 0, belongs to the induced subset 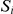. By analogy, constraints (1.11) force the 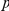-th site of the haplotype 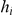 to be equal to 1 when at least one genotype 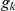, whose 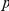-th entry equal to 2, belongs to the induced subset 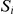. Constraints (1.12–1.13) force one of the two 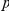-th sites of haplotypes 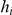 and 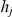 to be equal to 1 when the 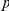-th entry of genotype 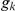 is equal to 1. Constraints (1.14–1.17) are the analogous version of constraints (1.10–1.13) in presence of uncertainty in the input data. Constraints (1.18–1.20) impose that at most one variable 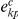 can be equal to one in presence of uncertainty in the input data. Finally, constraints (1.21–1.22) impose the upper and lower bounds on the error variables 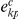.

### Reducing model size

The particular nature of the set of indices 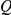 can be exploited to reduce the size of the CRM for PPH-UD. This operation proves fundamental to vastly improve the efficiency of whole model. Specifically, as shown in Catanzaro *et al.* [1], variables 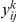 belonging to one of the following sets:
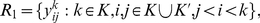
(2)

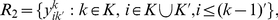
(3)

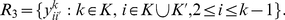
(4)do not need to be defined. Moreover, the sets of redundant variables can be further expanded by taking into account the entries of the error mask matrix and by observing that for each triplet of genotypes 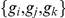 such that the respective 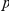-th SNP is 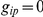, 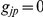, 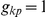, and 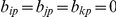, variable 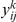 is necessarily equal to 0 since the containment of genotype 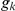 to the subsets 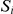 and 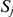 would violate the sum operator among haplotypes at least at 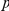-th SNP. Extending this argument to all the possible combinations of triplets of genotypes that violate the haplotype sum operator, it is easy to see that the following sets of variables are redundant and can be removed from the model:
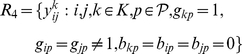
(5)

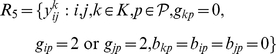
(6)

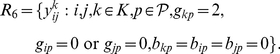
(7)Note that, removing the redundant variables 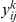 belonging to the sets 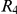
–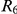 can be performed in 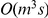. Finally, a similar process of reduction can be applied to variables 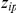 both by removing those whose value is fixed by constraints (1.6) (e.g., when 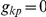 or when 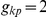). In this way, only variables 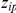 involved in constraints (1.6) when 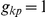 and in constraints (1.7–1.9) need to be defined.

## Results and Discussion

In this section we analyze the performances of the Class Representative Model (CRM) to solve instances of the pure parsimony haplotyping problem under uncertain data. Similar to Brown and Harrower [30] and Catanzaro *et al.* [1], we emphasize that our experiments aim simply to evaluate the runtime performance of our model for solving PPH. We neither attempt to study the efficiency of PPH for haplotype inference nor compare the accuracy of our algorithm to haplotype inference solvers that do not use the parsimony criterion. This analysis has been already performed by Gusfield [31], Wang and Xu [28], and Marchini *et al.* [32], and we refer the interested reader to their respective articles.

As in Catanzaro *et al.* [1], we used the standard Brown and Harrower's datasets [30] for testing the performances of our model. Specifically, through Hudson's MS program [33], Brown and Harrower created two families of datasets (called the *uniform* and *nonuniform* datasets) by randomly pairing the resulting haplotypes. The distinction in the two simulated methods comes in how the random pairing is performed. In the uniform datasets the haplotypes are randomly paired by sampling uniformly from the set of distinct haplotypes. In the nonuniform datasets the haplotypes are sampled uniformly from the collection of haplotypes generated by the coalescent process. In this collection, haplotypes may not be unique, so some haplotypes are sampled with higher frequency than others. Both the uniform and non-uniform datasets consist of collections of 30 or 50 genotypes having 10, 30, 50, 75 or 100 SNPs each. Each dataset contains a number of instances variable between 15 and 50. The authors also considered biological data from chromosomes 10 and 21, over all four Hap-Map [21] populations. For each input the authors selected sequences having 30, 50, and 75 SNPs, respectively, giving a total of 8 datasets consisting of 3 instances each. Brown and Harrower's datasets are not subjected to uncertainty, for this reason we considered four sets of random generated error mask matrices having an error ratio (i.e., the number of entries equal to 1 divided 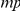) equal to 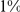, 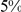, 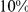, and 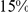 respectively. Brown and Harrower's datasets and the error mask matrices used in our experiments can be downloaded at the address: http://homepages.ulb.ac.be/~dacatanz/PPHerr.zip.

In Tables 2–5 we show the performances of the CRM for PPH-UD under different error ratios by showing, conservatively, the same information described in Brown and Harrower [30] and Catanzaro *et al.* [1]. Specifically, the columns of Tables 2–5 evidence the average, the maximum, and the minimum of: the solution time, the gap (i.e., the difference between the optimal value found and the value of linear relaxation at the root node of the search tree, divided by the optimal value), and the number of nodes expanded in each group of instances belonging to a given dataset. The results have been obtained by implementing the CRM for PPH-UD in Mosel 2.0 of Xpress-MP, Optimizer version 18, running on a Pentium 4, 3.2 GHz, equipped with 2 GByte RAM and operating system Gentoo release 7 (kernel linux 2.6.17). In our experiments we activated the Xpress-MP Optimizer automatic cuts, the Xpress-MP pre-solving strategy, and used the Xpress-MP primal heuristic to generate the first upper bound.

**Table 2:** Performances of the CRM for PPH-UD when considering input data having an error ratio of 1%.

Dataset	Time (sec.)			Gap (%)			Nodes		
	Average	Max	Min	Average	Max	Min	Average	Max	Min
**Uniform**									
50×10	9.919	34.771	2.381	0.000	0.000	0.000	1.000	1	1
50×10r4	11.446	37.582	2.899	0.000	0.000	0.000	1.000	1	1
50×10r16	12.247	31.963	3.144	0.556	8.333	0.000	1.467	7	1
50×30	43.089	94.633	11.440	0.743	5.882	0.000	5.867	42	1
30×50	35.471	130.316	4.053	1.569	9.091	0.000	34.490	531	1
30×75	57.063	211.341	9.842	1.475	6.250	0.000	42.000	159	1
30×100	85.675	254.772	21.987	0.285	2.778	0.000	30.222	186	3
**Non-Uniform**									
50×10	10.601	88.367	1.857	8.333	0.000	0.000	1.000	1	0
50×30	41.606	134.018	4.833	0.401	5.882	0.000	18.200	81	1
30×50	30.277	89.376	1.531	1.144	4.785	0.000	60.400	231	1
30×75	98.110	235.745	25.101	0.410	3.947	0.000	175.000	1039	1
30×100	624.549	7209.090	44.103	0.866	4.545	0.000	2290.667	31284	17
**Biological**									
CHR10-CEU	35.717	81.623	0.465	1.667	5.000	0.000	124.000	341	1
CHR21-CEU	8.811	23.387	0.106	0.000	0.000	0.000	2.000	3	1
CHR10-HBC	111.930	308.471	0.000	2.381	7.143	0.000	800.667	1750	0
CHR21-HBC	33.311	99.340	0.069	5.556	16.667	0.000	29.667	83	1
CHR10-JPT	16.022	46.252	0.758	1.458	4.375	0.000	41.667	121	1
CHR21-JPT	14.004	19.798	8.209	0.018	0.035	0.000	39.500	76	3
CHR10-YRI	2491.275	7245.020	47.468	1.562	2.652	0.034	5477.333	16323	53
CHR21-YRI	2548.248	7641.350	0.459	2.667	8.000	0.000	143.667	429	1

**Table 3 pone-0017937-t003:** Performances of the CRM for PPH-UD when considering input data having an error ratio of 5%.

Dataset	Time (sec.)			Gap (%)			Nodes		
	Average	Max	Min	Average	Max	Min	Average	Max	Min
**Uniform**									
50×10	9.775	35.814	2.030	0.000	0.000	0.000	1.000	1	1
50×10r4	8.699	62.160	2.642	0.000	0.000	0.000	1.000	1	1
50×10r16	11.055	44.405	2.635	0.000	0.000	0.000	2.067	7	1
50×30	43.273	122.443	7.878	1.338	5.882	0.000	5.533	49	1
30×50	36.903	178.408	4.270	1.620	9.091	0.000	34.760	827	1
30×75	40.422	115.258	10.835	1.383	6.250	0.000	9.900	37	1
30×100	98.348	354.685	7.330	0.484	2.590	0.000	43.700	244	1
**Non-Uniform**									
50×10	12.878	135.199	1.576	1.000	8.333	0.000	1.000	1	1
50×30	42.623	193.406	3.422	0.498	4.762	0.000	14.667	111	1
30×50	32.124	120.886	1.576	0.837	4.762	0.000	62.467	562	1
30×75	110.105	323.692	29.245	0.644	3.819	0.000	203.200	1317	3
30×100	639.736	7210.800	45.658	0.642	4.000	0.000	2527.867	34214	6
**Biological**									
CHR10-CEU	25.568	76.186	0.033	5.000	5.000	0.000	83.667	249	1
CHR21-CEU	12.137	32.109	0.593	0.000	0.000	0.000	16.333	27	1
CHR10-HBC	54.896	150.916	1.570	2.381	7.143	0.000	121.000	321	1
CHR21-HBC	33.719	100.581	0.071	10.317	16.667	0.000	9.667	23	1
CHR10-JPT	5.204	14.500	0.003	1.668	5.000	0.000	5.000	13	1
CHR21-JPT	9.691	19.591	0.021	1.830	5.490	0.000	43.667	123	1
CHR10-YRI	2864.727	7254.880	49.311	0.889	2.667	0.000	5464.000	13956	77
CHR21-YRI	2551.640	7651.670	0.165	8.684	26.051	0.000	128.000	382	1

**Table 4 pone-0017937-t004:** Performances of the CRM for PPH-UD when considering input data having an error ratio of 10%.

Dataset	Time (sec.)			Gap (%)			Nodes		
	Average	Max	Min	Average	Max	Min	Average	Max	Min
**Uniform**									
50×10	9.641	47.717	2.461	0.000	0.000	0.000	1.000	1	1
50×10r4	16.057	62.160	2.663	0.000	0.000	0.000	1.067	2	1
50×10r16	13.838	44.178	2.678	0.000	0.000	0.000	2.667	9	1
50×30	38.338	85.872	9.874	1.445	8.889	0.000	6.733	35	1
30×50	39.799	222.460	3.249	1.192	8.333	0.000	40.020	1113	1
30×75	43.441	138.641	10.366	1.579	6.250	0.000	22.800	58	1
30×100	120.666	323.663	17.943	0.303	2.778	0.000	78.800	538	1
**Non-Uniform**									
50×10	13.174	126.922	1.765	8.333	0.000	0.000	1.000	1	0
50×30	40.194	84.860	2.418	0.919	5.882	0.000	23.933	265	1
30×50	27.455	73.425	1.488	0.922	4.737	0.000	18.467	66	1
30×75	108.737	325.864	33.529	0.814	4.348	0.000	250.733	1539	3
30×100	1563.970	7208.110	37.634	0.773	4.000	0.000	11673.800	74593	2
**Biological**									
CHR10-CEU	32.592	95.712	0.209	0.000	0.000	0.000	144.667	431	1
CHR21-CEU	4.935	11.259	0.544	1.852	5.556	0.000	2.000	4	1
CHR10-HBC	185.619	529.879	2.228	2.381	7.143	0.015	1739.333	4926	1
CHR21-HBC	42.578	127.162	0.074	10.317	16.667	0.000	33.000	93	1
CHR10-JPT	19.037	56.795	0.003	1.667	5.000	0.000	33.667	99	1
CHR21-JPT	8.433	19.635	2.716	2.225	6.667	0.000	33.667	99	1
CHR10-YRI	2992.214	7231.800	30.751	0.877	2.632	0.000	6538.000	14597	9
CHR21-YRI	2534.816	7600.780	1.083	4.119	12.356	0.000	117.000	349	1

**Table 5:** Performances of the CRM for PPH-UD when considering input data having an error ratio of 15%.

Dataset	Time (sec.)			Gap (%)			Nodes		
	Average	Max	Min	Average	Max	Min	Average	Max	Min
**Uniform**									
50×10	10.092	57.006	1.560	0.000	0.000	0.000	1.000	1	1
50×10r4	11.570	33.895	3.275	0.000	0.000	0.000	1.000	1	1
50×10r16	9.209	18.627	2.790	1.068	8.333	0.000	1.467	5	1
50×30	44.276	157.222	7.467	1.123	5.882	0.000	4.133	15	1
30×50	39.772	222.039	6.187	1.006	8.333	0.000	42.700	1149	1
30×75	49.558	160.645	8.048	1.409	6.250	0.000	19.500	67	1
30×100	97.879	262.453	23.986	0.664	3.836	0.000	32.800	98	1
**Non-Uniform**									
50×10	9.925	126.922	2.113	1.000	8.333	0.000	1.133	1	1
50×30	38.689	84.860	4.000	0.400	5.882	0.000	11.267	265	1
30×50	33.272	73.425	1.793	0.614	4.737	0.000	54.400	66	1
30×75	88.030	325.864	32.260	0.800	4.348	0.000	95.400	1539	3
30×100	631.495	7207.900	50.928	1.157	4.270	0.000	2371.200	32622	12
**Biological**									
CHR10-CEU	27.340	68.299	1.669	0.000	0.000	0.000	53.667	155	3
CHR21-CEU	13.455	36.601	0.522	0.000	0.000	0.000	6.667	12	1
CHR10-HBC	87.770	248.825	2.228	4.347	7.143	0.015	384.333	1127	1
CHR21-HBC	39.878	118.866	0.075	10.317	16.667	0.000	27.000	69	1
CHR10-JPT	23.436	69.457	0.002	1.667	5.000	0.000	72.667	213	1
CHR21-JPT	2403.091	7188.610	2.781	2.222	6.667	0.000	4406.333	13125	1
CHR10-YRI	702.533	1777.450	62.414	0.000	0.000	0.000	583.000	1635	48
CHR21-YRI	2545.198	7630.880	1.500	5.449	16.346	0.000	123.333	368	1

In order to obtain a qualitative measure of the running time performances of the CRM for PPH-UD, we compared the numerical results of the model with the corresponding ones of the CRM for PPH (RM version, see Catanzaro *et al.* [1]) running on the same datasets in absence of uncertainty. The performances of the CRM for PPH are shown in Table 6. Moreover, in order to obtain a measure of the accuracy of the CRM for PPH-UD, we used the following procedure: fixed a generic instance of PPH-UD, we computed the optimal solution provided by CRM for PPH in absence of uncertainty and considered the corresponding set of haplotypes as the “correct set”; subsequently, we computed the optimal solution provided by CRM for PPH-UD in presence of uncertainty (i.e., when taking into account the corresponding input error mask matrix) and assumed, as measure of the accuracy, the ratio between the number of equal haplotypes in both solutions divided the overall number of haplotypes in the solution provided by CRM for PPH. When such a ratio is equal to 1 it means that CRM for PPH-UD was able to recover the correct haplotype set, otherwise, when the ratio is smaller than 1 it means that CRM for PPH-UD was able to recover only a fraction of such a set. The accuracy (expressed in percentage) of the CRM for PPH-UD under increasing error ratios is shown in Table 7. For sake of notation, in the following subsections we shall denote CRM1 and CRM2 as the CRM for PPH and the CRM for PPH-UD, respectively.

**Table 6:** Performances of the CRM for PPH (RM version) on Brown and Harrower's datasets [30].

Dataset	Time (sec.)			Gap (%)			Nodes		
	Average	Max	Min	Average	Max	Min	Average	Max	Min
**Uniform**									
50×10	1.143	2.404	0.102	0.000	0	0	1.000	1	1
50×10r4	1.730	6.104	0.043	1.179	10	0	1.000	1	1
50×10r16	8.092	30.623	2.011	1.644	10.7692	0	1.533	9	1
50×30	11.772	53.42	2.732	2.440	7.14286	0	2.000	15	1
30×50	8.922	47.467	0.73	1.694	7.69231	0	10.260	75	1
30×75	15.624	35.693	1.358	1.649	6.66667	0	24.300	92	1
30×100	10.142	31.994	2.593	1.402	7.35294	0	8.500	25	1
**Non-Uniform**									
50×10	0.634	1.726	0.127	0.513	7.69231	0	2.400	11	1
50×30	11.882	30.411	1.59	1.164	6.25	0	11.867	35	1
30×50	10.764	24.108	0.815	0.890	4.09091	0	20.533	61	1
30×75	22.389	61.869	3.537	1.038	5.55556	0	62.286	387	1
30×100	74.925	462.791	12.953	1.521	4.7619	0	216.071	1679	8
**Biological**									
CHR10-CEU	102.792	305.103	0.774	0.000	0	0	270.333	807	1
CHR21-CEU	18.868	54.562	0.428	1.515	4.54545	0	49.667	145	1
CHR10-HBC	38.058	96.324	8.746	2.593	7.77778	0	67.000	151	1
CHR21-HBC	0.182	0.456	0.017	0.000	0	0	8.000	19	1
CHR10-JPT	0.895	1.583	0.368	1.515	4.54545	0	7.000	11	1
CHR21-JPT	1.781	2.87	0.967	0.833	2.5	0	15.667	29	1
CHR10-YRI	73.723	116.127	31.353	1.111	3.33333	0	89.667	123	63
CHR21-YRI	2349.331	6819.2	50.012	0.000	0	0	3815.667	11199	123

**Table 7:** Accuracy of the CRM for PPH-UD under different error ratios.

Dataset	1(%)			5(%)			10(%)			15(%)		
	Average	Max	Min	Average	Max	Min	Average	Max	Min	Average	Max	Min
**Uniform**												
50×10	100.00	100.00	100.00	99.01	100.00	92.31	100.00	100.00	100.00	98.02	100.00	90.91
50×10r4	100.00	100.00	100.00	99.51	100.00	88.89	99.02	100.00	88.89	99.51	100.00	88.89
50×10r16	99.11	100.00	92.31	98.22	100.00	84.62	98.22	100.00	92.31	96.00	100.00	75.00
50×30	98.82	100.00	92.86	96.85	100.00	78.57	92.91	100.00	70.59	94.88	100.00	71.43
30×50	95.68	100.00	57.14	90.22	100.00	57.14	89.83	100.00	50.00	84.24	100.00	25.00
30×75	96.59	100.00	81.25	88.07	100.00	62.50	86.36	100.00	62.50	77.84	100.00	6.25
30×100	96.59	100.00	81.25	94.89	100.00	82.35	90.91	100.00	75.00	90.34	100.00	64.71
**Non-Uniform**												
50×10	95.07	100.00	81.25	92.61	100.00	78.57	96.55	100.00	86.67	94.58	100.00	81.25
50×30	92.38	100.00	82.35	88.41	100.00	64.71	89.40	100.00	64.71	86.42	100.00	64.71
30×50	88.55	100.00	63.16	86.87	100.00	68.42	85.52	100.00	68.75	81.14	100.00	59.09
30×75	85.50	100.00	60.00	80.97	100.00	56.52	79.46	100.00	66.67	80.97	100.00	56.52
30×100	76.72	100.00	68.00	82.18	95.00	72.00	75.00	95.71	65.00	78.74	95.71	60.00
**Biological**												
CHR10-CEU	89.39	100.00	80.00	83.33	100.00	70.83	78.79	86.36	70.83	68.18	80.00	54.17
CHR21-CEU	93.75	100.00	83.33	91.67	100.00	83.33	58.33	75.00	33.33	56.25	83.33	27.78
CHR10-HBC	78.05	90.00	64.71	68.29	92.86	23.53	53.66	85.71	23.53	51.22	80.00	17.65
CHR21-HBC	99.84	100.00	73.68	99.81	100.00	68.42	65.63	100.00	47.37	71.88	100.00	52.63
CHR10-JPT	80.95	100.00	72.73	71.43	100.00	55.00	66.67	90.91	45.00	59.52	100.00	30.00
CHR21-JPT	84.91	94.12	66.67	69.81	80.95	60.00	50.94	57.14	41.18	43.40	52.94	33.33
CHR10-YRI	73.26	76.00	69.44	66.28	80.00	52.78	53.49	84.00	30.56	48.84	68.00	25.00
CHR21-YRI	63.29	100.00	50.94	50.63	100.00	35.85	41.77	100.00	20.75	40.51	100.00	26.42

### Uniform Datasets

The experiments relative to the uniform datasets showed that, when considering an error ratio of 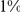 already, CRM2 takes significantly more time than CRM1 to solve Brown and Harrower's datasets, confirming the hardness of PPH-UD with respect to PPH. Specifically, Tables 2–5 show, as general trend, that the higher the error ratio the slower the runtime performances of the model. For example, while CRM1 took in average 8 seconds to solve the most difficult dataset having 10 SNPs, CRM2 took in average at least 10 seconds independently from the error ratio, and even longer on instances 03, 05, 06, 08 and 11 of dataset 50×10r4 where 19.657, 62.160, 34.429, 40.416, and 15.974 seconds, respectively, were needed to find the optimum. This trend persists also in the instances having 30 SNPs, where CRM1 took in average 11.772 seconds while CRM2 needed an average solution time of 43.273 seconds when considering an error ratio of 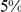, with the exception of instances 02, 08, 09, 11, 13 and 14 which needed 62.182, 51.462, 60.079, 60.020, 122.443, and 58.514 seconds, respectively. We observed that the overall performances of CRM2 with an error ratio of 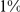 generally tend to be similar to the ones of CRM2 with an error ratio of 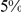; moreover, we experienced also a generalized decrement of the average solution time when considering an error ratio of 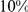 and, vice versa, an increment of the average solution time when considering an error ratio of 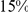. When considering instances having a larger number of SNPs, we experienced a generalized increment of the average solution time taken by CRM2, proportional to the increment of the error ratio. Interestingly, the average gap and number of branches performed by CRM2, although not directly comparable with the corresponding one of CRM1, results relatively small, confirming the tightness of the class representative model also for uncertain data.

The accuracy of CRM2 in the uniform datasets result very good. Specifically, the average accuracy is over 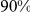 in the majority of the analyzed datasets and independently from the error ratio. However, it is worth noting that in some instances the accuracy may decrease significantly (see, e.g., datasets 30×50 and 30×75) and proportionally to the increment of the error ratio, by suggesting, as general trend, the fact that the higher the error ratio the more difficult is to recover the correct haplotype set.

### Nonuniform Datasets

The general trend observed in the uniform datasets persists also in the nonuniform datasets. Specifically, as shown in Table 4, CRM2 took in average 10 times more the average solution time taken by CRM1 to solve instances having 10 SNPs, reaching a maximum solution time of 135.199 seconds when tackling instance 06 affected by an error ratio of 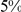. Similarly, when tackling instances having 30 SNPs, CRM2 took in average 4 times more the average solution time taken by CRM1, reaching a maximum solution time of 193.406 seconds when tackling instance 00 affected by an error ratio of 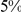. When dealing with instances having more than 30 SNPs, CRM2 took significantly more than CRM1 reaching a solution time of 7210.800 seconds when tackling the instance 100-30.03 affected by an error ratio of 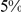.

The accuracy of CRM2 in the nonuniform datasets result still good, but slightly poorer than in the uniform datasets. Specifically, the average accuracy is over 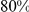 in the majority of the analyzed datasets and independently from the error ratio. Similarly to the uniform datasets, in some instances the accuracy may decrease significantly (see, e.g., datasets 30×75). However, in the worst case, the decrement results much smaller than the corresponding one in the uniform datasets.

### Biological Datasets

To complete the performance analysis on Brown and Harrower's datasets, we tested CRM2 on the biological datasets. Once again, the general trend observed in the uniform and nonuniform datasets persists also in the biological datasets: CRM2 results significantly slower than CRM1, a part from datasets CHR10-CEU and CHR21-CEU in which the trend is inverted due to the peculiar nature of both datasets. While the average gap of CRM1 never exceeded 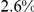, the average gap of CRM2 was 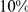 or more, confirming the hardness of the biological datasets. However, we stress once again the fact that PPH and PPH-UD are de facto two different problems, hence intrinsic values such as the gap cannot be directly compared. Our analysis just aims at offering experimental evidence of the tightness of the class representative model in tackling instances of the pure parsimony haplotyping problem under uncertain data.

The small number of instances constituting each biological dataset (three instances per dataset) prevents a clear statistical characterization of the performances of CRM2 in terms of accuracy. As general trend, we have observed that the accuracy approaches 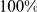 in the majority of the biological datasets analyzed. Nevertheless, in a number of datasets this trend changes, leading the accuracy level to low values. Investigating the reason why this phenomenon arises and the possible corresponding remedies warrants additional analysis.

### Conclusion

In this article we have investigated, for the first time, a recent version of PPH, called the Pure Parsimony Haplotype problem under Uncertain Data (PPH-UD). This version mainly arises when the input genotypes are not accurate, i.e., when some single nucleotide polymorphisms are missing or affected by errors. We proposed an exact approach to solution of PPH-UD based on an extended version of Catanzaro *et al.* [1] class representative model for PPH, possibly one of the best integer programming models described so far in the literature on PPH. The model is efficient, accurate, compact, polynomial-sized, easy to implement, solvable with any solver for mixed integer programming, and usable in all those cases for which the parsimony criterion is well suited for haplotype estimation.
